# Editorial for the Special Issue “Advances in Phenolic Based Complexes”

**DOI:** 10.3390/molecules31152593

**Published:** 2026-07-24

**Authors:** Kiril Gavazov

**Affiliations:** Department of Chemical Sciences, Faculty of Pharmacy, Medical University of Plovdiv, 120 Buxton Bros Str., 4004 Plovdiv, Bulgaria; kiril.gavazov@mu-plovdiv.bg

**Keywords:** phenolic compounds, metal complexes, coordination chemistry, photoisomerization, nanoparticles, green chemistry, biological activity, therapeutic applications, analysis, (poly)phenols

## Abstract

Research on phenolic compounds and their complexes continues to advance rapidly, driven by interdisciplinary efforts that expand both their fundamental scientific significance and practical applications. This Special Issue of *Molecules* presents recent developments in the chemistry and applications of these complexes through contributions from researchers across three continents. The published papers can be organized into five thematic sections: (i) synthesis and structural characterization; (ii) biological activity and therapeutic potential; (iii) analytical applications and sensors; (iv) nanotechnology and advanced delivery systems; and (v) metabolism and environmental factors. Among the contributions is a review article on the application of phenolic azobenzene ligands for cation complexation. Collectively, the eight contributions provide an up-to-date overview of the field, highlighting recent advances, emerging trends, innovative approaches, and future research directions in the chemistry and applications of phenolic-based complexes.

## 1. Introduction

Phenolic compounds constitute a diverse class of aromatic molecules characterized by one or more hydroxyl groups directly attached to an aromatic ring. As weak acids, they readily undergo deprotonation to form phenoxide ions under alkaline conditions, while their susceptibility to oxidation leads to the formation of quinones—highly reactive intermediates involved in numerous chemical and biological processes. The electron-donating effect of the hydroxyl group increases the electron density of the aromatic ring, thereby enhancing its reactivity and enabling a wide range of chemical transformations.

Owing to their structural diversity and versatile coordination behavior, phenolic compounds have attracted considerable interest in coordination chemistry, analytical chemistry, supramolecular chemistry, and materials science [1,2,3,4,5,6,7]. Depending on the number and relative positions of their hydroxyl groups, they can be broadly classified into monohydric phenols, phenols with isolated hydroxyl groups, ortho-hydroxylated phenols (catechol derivatives), peri-hydroxylated phenols, multifunctional phenolic derivatives, and polyphenols. Their ability to coordinate metal ions through different binding modes has led to the development of a wide variety of metal complexes, with applications ranging from chemical sensing to catalysis and biomedicine [6,7,8,9,10,11,12].

A key aspect of progress in these materials is the development of multifunctional ligands, such as phenolic azobenzene derivatives. These molecules have the ability to form complexes with metal cations and possess the unique property of the azobenzene core for reversible photoisomerization. This combination makes them perfect for creating “smart” materials and colorimetric sensors that can detect heavy metals [13,14,15,16]. Similarly, catechol-based ligands are of significant interest due to their affinity for transition metal ions. This feature allows for their use in metal ion determination following environmentally friendly preconcentration procedures [17,18,19,20,21].

The incorporation of phenolic compounds and their metal complexes into nanotechnology has further broadened their range of applications. Plant- and macrofungi-derived phenolic extracts have been successfully employed in the “green synthesis” of metal nanoparticles [22,23,24,25,26], where natural phenolic metabolites function simultaneously as reducing and stabilizing agents. In parallel, the encapsulation of phenolic compounds in chitosan-based nanocomposites or nanoemulsion systems has been shown to improve their solubility, stability, bioavailability, and controlled release, thereby enhancing their antioxidant, antimicrobial, and other biological activities [27,28,29,30,31,32].

The application potential of phenolic-based complexes continues to expand across numerous scientific disciplines. In materials science, they contribute to the development of functional materials and the optimization of dyes for dye-sensitized solar cells, whereas in the biomedical field, metal complexes of phenolic compounds have demonstrated considerable promise as antimicrobial, gastroprotective, antidiabetic, antioxidant, and anticancer agents [1,2,3,11]. At the same time, advances in analytical chemistry have enabled the development of sensitive, simple, robust, and environmentally sustainable methods for metal ion determination [33,34], while studies on the metabolism and bioavailability of dietary phenolics are improving our understanding of their health-promoting effects [35,36,37,38,39,40].

The aim of this Special Issue (SI) was to provide an international forum for presenting recent advances in the synthesis, structural characterization, physicochemical properties, biological activities, analytical applications, and emerging technological uses of phenolic-based complexes. The eight contributions included in this Special Issue illustrate the broad scope of current research in this rapidly evolving field, highlighting innovative developments in coordination chemistry, nanotechnology, analytical science, and biomedicine while emphasizing the growing importance of phenolic compounds in addressing contemporary scientific and technological challenges.

## 2. Thematic Sections

The contributions to this SI can be grouped into five major thematic sections that highlight recent advances in the synthesis, analytical utility, biomedical potential, and environmental aspects of these complexes.

### 2.1. Synthesis and Structural Features

This section encompasses studies focused on the design, synthesis, and structural characterization of phenolic ligands and their metal complexes.

- Phenolic azobenzenes as ligands: Novel phenolic azobenzene derivatives capable of reversible photoisomerization and coordination with metal cations have been synthesized and investigated, demonstrating their potential as photoresponsive ligands [Contribution 1].
- Metal complexes of curcuminoids: Copper complexes of curcumin analogues containing an extended unsaturated chain have been synthesized and structurally characterized using X-ray crystallography and EPR spectroscopy [Contribution 2].
- Iron coordination with 4-nitrocatechol: A stable low-spin Fe(III) complex with 4-nitrocatechol has been obtained, and its molecular structure has been elucidated through quantum-chemical calculations, supported by spectrophotometric data [Contribution 3].

### 2.2. Biological Activity and Therapeutic Potential

A substantial proportion of the contributions investigate the biological properties of phenolic-based complexes and their potential biomedical applications.

- Antibacterial and anti-urease activity: Metal complexes of diacetylcurcumin with Cu(II), Zn(II), Mn(II), and Mg(II) exhibit significant inhibitory activity against Helicobacter pylori and its urease enzyme, highlighting their promise as antimicrobial agents [Contribution 4].
- Antidiabetic and antiproliferative properties: Copper complexes of curcuminoids, including β-cyclodextrin-associated systems, demonstrate considerable inhibitory activity toward β-glucosidase, indicating potential applications in diabetes management and oncology [Contribution 2].
- Broad-spectrum biological activity: Phenolic compounds derived from natural products (in particular macrofungi) display diverse biological effects, including antioxidant, anti-inflammatory, and antitumor activities. Additionally, their ability to chelate transition metals contributes to mitigating the formation of reactive oxygen species [Contribution 5].

### 2.3. Analytical Applications and Chemical Sensors

This section showcases the utility of phenolic complexes in analytical chemistry and sensing technologies.

- Colorimetric sensors: Hydroxyazobenzene derivatives function as selective colorimetric chemosensors by undergoing distinct color changes upon binding to metal ions such as Cu(II), Cr(III), and Hg(II) [Contribution 1].
- Spectrophotometric analysis: A semi-micro extraction–spectrophotometric method has been developed for the sensitive determination of Fe(III). The method has been successfully implemented in the analysis of pharmaceutical formulations and industrial samples [Contribution 3].

### 2.4. Nanotechnology and Advanced Delivery Systems

This section highlights recent advances in improving the stability, bioavailability, and functional performance of phenolic compounds through nanotechnology-based approaches.

- Chitosan composites and nanoemulsions: Chitosan-based composites incorporating nanoemulsions of essential oils, such as Artemisia argyi, have been developed to enhance physicochemical stability, antioxidant capacity, and the controlled release of bioactive compounds [Contribution 6].
- Green synthesis of nanoparticles: Plant-derived phenolic compounds, including those from duckweed (*Lemna* spp.), have been utilized as reducing and stabilizing agents for the environmentally friendly synthesis of zinc oxide nanoparticles (ZnO NPs), thereby improving their biological performance [Contribution 7].

### 2.5. Metabolism and Environmental Influences

This section explores the factors affecting the metabolism of phenolic compounds and the influence of environmental conditions on their biosynthesis.

- Phenolic metabolism in metabolic syndrome: Studies investigating the metabolism of beer-derived phenolic compounds demonstrate that metabolic syndrome significantly influences the formation of bioactive conjugates and microbial catabolites, emphasizing the role of individual physiological status in phenolic bioavailability [Contribution 8].
- Effects of salt stress: Salt stress has been shown to alter the phenolic composition of plants by increasing flavonoid accumulation, which subsequently modulates the physicochemical characteristics and biological properties of the nanoparticles synthesized from these plant extracts [Contribution 7].

## 3. An Overview of the Individual Contributions

Leonard et al. [Contribution 1] presented a comprehensive review highlighting phenolic azobenzenes as multifunctional compounds with considerable potential in coordination chemistry. The authors summarize the principal synthetic strategies for these molecules, including azo coupling reactions and the oxidative coupling of anilines, while discussing the influence of substituent effects on reaction efficiency and product yields.

A major focus of the review is the ability of phenolic azobenzenes to function as ligands for metal cations, with particular emphasis on the crucial role of the phenolic hydroxyl group in metal coordination and complex stabilization. The review further examines the distinctive photochemical properties of these compounds, especially their reversible trans–cis photoisomerization upon light irradiation.

The combination of efficient metal-binding properties and reversible photoswitching makes phenolic azobenzenes attractive building blocks for a broad range of functional applications, including colorimetric and optical sensors, molecular switches, catalysts, and sensitizers for dye-sensitized solar cells. Overall, the review demonstrates that rational structural design of phenolic azobenzenes enables fine tuning of their coordination behavior, photophysical properties, and chemical reactivity, thereby broadening their applicability in advanced functional materials and molecular devices.

Tavera-Hernández et al. [Contribution 2] reported the synthesis and comprehensive characterization of novel curcuminoid analogues featuring an extended 11-carbon π-conjugated chain, together with their boron and copper complexes. The study is particularly noteworthy for describing the first Cu(II) complex of a C=C-extended curcuminoid analogue and investigating its structural, supramolecular, and biological properties.

The synthesized compounds were thoroughly characterized using a combination of spectroscopic methods, including NMR spectroscopy, and single-crystal X-ray diffraction analysis. These techniques confirmed their molecular structures and provided detailed insight into their structural features and optical properties.

Biological evaluation revealed that the synthesized compounds exhibit significant inhibitory activity against α-glucosidase, highlighting their potential as lead candidates for the development of novel therapeutics for type 2 diabetes mellitus. Overall, the study demonstrates that the combination of metal complexation and supramolecular encapsulation represents an effective strategy for enhancing the physicochemical and biological properties of curcuminoid derivatives. In particular, the newly synthesized copper complex emerges as a promising bioactive compound with favorable biological activity and a potentially advantageous safety profile.

Racheva et al. [Contribution 3] developed an innovative semi-micro extraction–spectrophotometric procedure for the determination of Fe(III) based on the formation of a ternary ion-association complex with 4-nitrocatechol and xylometazoline hydrochloride. The authors systematically optimized the experimental conditions to achieve high sensitivity, selectivity, and precision. The composition and structure of the extracted complex were elucidated through a combination of experimental investigations and quantum-chemical calculations performed at the B3LYP/6-311G level of theory.

The proposed procedure provides a simple, sensitive, and cost-effective approach for iron determination. In addition, it requires only small volumes of organic solvent, thereby reducing solvent consumption and making the procedure more environmentally friendly and consistent with the principles of green analytical chemistry.

Agabo-Martínez et al. [Contribution 4] investigated the therapeutic potential of diacetylcurcumin (DAC) and its metal complexes as alternative agents for the treatment of Helicobacter pylori infections, a major cause of gastritis, peptic ulcers, and other gastric disorders. The study evaluated the antibacterial activity, urease inhibitory effects, and gastroprotective properties of DAC complexes containing different metal ions.

Among the investigated compounds, the Cu(II) and Zn(II) complexes exhibited the strongest antibacterial activity against *H. pylori* and effectively inhibited urease, a key virulence factor that enables the bacterium to survive in the highly acidic gastric environment. Furthermore, in vivo studies in a mouse model demonstrated significant gastroprotective effects, with both complexes markedly reducing gastric mucosal damage.

The results indicate that metal coordination enhances the stability and biological activity of DAC while preserving a favorable safety profile. Overall, the study identifies DAC-based metal complexes, particularly the Cu(II) and Zn(II) derivatives, as promising candidates for the development of novel therapeutic strategies for the treatment of *H. pylori*-associated infections and gastric diseases.

Ildız and Canpolat [Contribution 5] investigated the biological properties of four species of wild edible macrofungi collected in Türkiye—*Tuber aestivum*, *Terfezia claveryi*, *Agaricus arvensis*, and *Bovistella utriformis*—with particular emphasis on their phenolic composition, antioxidant capacity, and antimicrobial activity. The study compared extracts obtained using solvents of different polarity and examined the relationship between phenolic content and biological activity.

The results demonstrated that methanolic extracts exhibited significantly higher antioxidant and antimicrobial activities than hexane extracts, reflecting the greater extraction efficiency of polar phenolic compounds. These extracts showed pronounced inhibitory effects, particularly against Gram-positive bacteria and multidrug-resistant bacterial strains. Among the investigated species, *B. utriformis* and *A. arvensis* contained the highest total phenolic contents and exhibited the strongest free radical scavenging activities.

Phytochemical analyses identified several bioactive phenolic compounds, including catechin and cinnamic acid, which are likely to contribute to the observed antioxidant and antimicrobial effects. Overall, the study highlights wild edible macrofungi as valuable natural sources of phenolic compounds with considerable potential for the development of functional foods, nutraceuticals, and novel antioxidant and antimicrobial agents.

Shi et al. [Contribution 6] presented an innovative strategy to enhance the therapeutic potential of *Artemisia argyi* essential oil, a natural product with well-established antibacterial and antioxidant properties but limited practical application due to its poor aqueous solubility, volatility, and chemical instability. To overcome these limitations, the authors developed a chitosan-based nanoemulsion system that serves as a stable carrier for the controlled release of the essential oil’s bioactive constituents.

Comprehensive physicochemical characterization confirmed the successful preparation of the nanocomposite and demonstrated its improved stability and encapsulation efficiency. Biological evaluation further revealed that the chitosan-based nanoemulsion significantly enhanced the antibacterial activity of the essential oil against resistant bacterial strains while preserving its antioxidant capacity. Compared with the free essential oil, the nanoformulation exhibited nearly a twofold increase in antibacterial efficacy, demonstrating the synergistic benefits of chitosan encapsulation and nanoemulsion technology.

In addition to its potential biomedical applications, the developed nanocomposite shows considerable promise for use in food preservation and cosmetic formulations, where improved stability and controlled release of bioactive compounds are highly desirable. Overall, the study demonstrates that chitosan-based nanoemulsion technology provides an environmentally friendly and effective platform for enhancing the stability, bioavailability, and biological performance of natural products, thereby expanding their potential applications in healthcare, food technology, and cosmetics.

Radulović et al. [Contribution 7] investigated the influence of salt stress on the phenolic profile of the aquatic plant *Lemna minor* (duckweed) and its subsequent effect on the green synthesis of zinc(II) oxide nanoparticles (ZnO NPs). The study demonstrated that increasing salinity during cultivation promoted the accumulation of phenolic compounds and flavonoids, which served as natural reducing and stabilizing agents during nanoparticle synthesis.

The synthesized ZnO NPs were comprehensively characterized to assess their structural and physicochemical properties. The results showed that the nanoparticles possessed a well-defined crystalline structure, while their crystallinity and phase composition were influenced by the level of salt stress applied to the source biomass. Furthermore, biological evaluation revealed that ZnO NPs synthesized from salt-stressed duckweed exhibited enhanced antioxidant activity and greater antimicrobial efficacy against *Staphylococcus haemolyticus* than those produced from non-stressed plants.

Overall, this study demonstrates how stress-induced metabolic changes in plants can be exploited to tailor the synthesis and functional properties of metal oxide nanomaterials. By linking alterations in the phenolic profile with nanoparticle characteristics and biological performance, the authors present an environmentally sustainable approach to producing bioactive ZnO NPs while promoting the valorization of aquatic biomass within the framework of a circular bioeconomy. These findings underscore the potential of plant-mediated green nanotechnology for applications in biomedicine, environmental remediation, and other advanced technological fields.

Hinojosa-Nogueira et al. [Contribution 8] investigated the metabolism of beer-derived (poly)phenols in individuals with and without metabolic syndrome through a controlled dietary intervention study. The work provides valuable insights into how physiological status influences the absorption, biotransformation, and metabolic fate of dietary phenolic compounds.

The results demonstrated that metabolic health plays a key role in shaping (poly)phenol metabolic profiles. Healthy participants exhibited greater production of specific phase II conjugates and flavonoid-derived metabolites, whereas individuals with metabolic syndrome showed increased formation of microbial-derived catabolites, indicating alterations in intestinal microbial metabolism. These differences suggest that metabolic disturbances associated with metabolic syndrome, including oxidative stress and gut microbiota dysbiosis, can significantly influence the biotransformation of beer-derived (poly)phenols.

Overall, this study highlights the complex interplay between dietary (poly)phenols, host metabolic status, and gut microbiota in determining the bioavailability and biological activity of phenolic metabolites. The findings emphasize the importance of considering individual physiological characteristics when evaluating the health effects of dietary phenolic compounds and support the growing concept of personalized nutrition based on metabolic and microbial profiles.

## 4. Conclusions

The eight contributions in this collection reflect the scope and diversity of contemporary research on phenolic compounds and highlight their growing scientific significance. Together, the studies demonstrate how phenolic compounds’ unique chemical properties continue to drive the development of innovative materials and technologies with broad application potential.

I express my sincere gratitude to all the authors for their high-quality contributions and unwavering dedication to advancing research in the field of phenolic complexes. I also thank the reviewers for their insightful and constructive evaluations and the *Molecules* editorial team for their support throughout the publication process. I hope that this SI will be a useful resource for researchers and encourage more investigations into chemistry, design, and uses of phenolic-based complexes.
